# Skeletal muscle oxygen saturation does not estimate mixed venous oxygen saturation in patients with severe left heart failure and additional severe sepsis or septic shock

**DOI:** 10.1186/cc5153

**Published:** 2007-01-16

**Authors:** Matej Podbregar, Hugon Možina

**Affiliations:** 1 Clinical Department for Intensive Care Medicine, University Clinical Centre, Zaloska 7, 1000 Ljubljana, Slovenia

## Abstract

**Introduction:**

Low cardiac output states such as left heart failure are characterized by preserved oxygen extraction ratio, which is in contrast to severe sepsis. Near infrared spectroscopy (NIRS) allows noninvasive estimation of skeletal muscle tissue oxygenation (StO_2_). The aim of the study was to determine the relationship between StO_2 _and mixed venous oxygen saturation (SvO_2_) in patients with severe left heart failure with or without additional severe sepsis or septic shock.

**Methods:**

Sixty-five patients with severe left heart failure due to primary heart disease were divided into two groups: groups A (*n *= 24) and B (*n *= 41) included patients without and with additional severe sepsis/septic shock, respectively. Thenar muscle StO_2 _was measured using NIRS in the patients and in 15 healthy volunteers.

**Results:**

StO_2 _was lower in group A than in group B and in healthy volunteers (58 ± 13%, 90 ± 7% and 84 ± 4%, respectively; *P *< 0.001). StO_2 _was higher in group B than in healthy volunteers (*P *= 0.02). In group A StO_2 _correlated with SvO_2 _(*r *= 0.689, *P *= 0.002), although StO_2 _overestimated SvO_2 _(bias -2.3%, precision 4.6%). In group A changes in StO_2 _correlated with changes in SvO_2 _(*r *= 0.836, *P *< 0.001; ΔSvO_2 _= 0.84 × ΔStO_2 _- 0.67). In group B important differences between these variables were observed. Plasma lactate concentrations correlated negatively with StO_2 _values only in group A (*r *= -0.522, *P *= 0.009; lactate = -0.104 × StO_2 _+ 10.25).

**Conclusion:**

Skeletal muscle StO_2 _does not estimate SvO_2 _in patients with severe left heart failure and additional severe sepsis or septic shock. However, in patients with severe left heart failure without additional severe sepsis or septic shock, StO_2 _values could be used to provide rapid, noninvasive estimation of SvO_2_; furthermore, the trend in StO_2 _may be considered a surrogate for the trend in SvO_2_.

**Trial Registration**: NCT00384644

## Introduction

Maintenance of adequate oxygen delivery (DO_2_) is essential to preservation of organ function, because sustained low DO_2 _leads to organ failure and death [1]. Low cardiac output states (cardiogenic, hypovolaemic and obstructive types of shock) and anaemic and hypoxic hypoxaemia are characterized by decreased DO_2 _but preserved oxygen extraction ratio. In distributive shock, the oxygen extraction capability is altered so that the critical oxygen extraction ratio is typically decreased [2]. Mixed venous oxygen saturation (SvO_2_), measured from the pulmonary artery, is used in the calculation of oxygen consumption and has been advocated as an indirect index of tissue oxygenation and a prognostic predictor in critically ill patients [3-6]. However, catheterization of the pulmonary artery is costly, has inherent risks and its usefulness remains subject to debate [7-9].

Near infrared spectroscopy (NIRS) is a technique that permits continuous, noninvasive, bedside monitoring of tissue oxygen saturation (Sto_2_) [9,10]. We previously showed that thenar muscle StO_2 _during stagnant ischaemia decreases at a slower rate in patients with septic shock than in patients with severe sepsis or localized infection and in healthy volunteers [11]. Patients included in the study had normal heart function and were haemodynamically stable; they also had normal or higher StO_2_. However, in every day clinical practice, we noticed extreme low levels of StO_2_, especially in patients with cardiogenic shock.

Our aim in the present study was to evaluate skeletal muscle oxygenation in severe left heart failure with or without additional severe sepsis/septic shock and to compare with with SvO_2_. The hypothesis was that StO_2 _may estimate SvO_2 _in patients severe left heart failure and preserved oxygen extraction capability (without severe sepsis/septic shock), because blood flowing through upper limb muscles could importantly contribute to flow through the superior vena cava. On the other hand, in patients with decreased oxygen extraction capability (with severe sepsis/septic shock), we expected disagreement between StO_2 _and SvO_2_, because in these patients greater oxygen extraction can probably take place in organs other than skeletal muscles.

## Materials and methods

### Patients

The study protocol was approved by the National Ethics Committee of Slovenia; informed consent was obtained from all patients or their relatives. The study was performed during the period between October 2004 and June 2006. Following initial heamodynamic resuscitation, heart examination was performed in all patients admitted to our intensive care unit (ICU) using transthoracic ultrasound (Hewlett-Packard HD 5000; Hewlett-Packard, Andover, MA, USA). In patients with primary heart disease, low cardiac output and no signs of hypovolaemia, right heart catheterization with a pulmonary artery floating catheter (Swan-Ganz CCOmboV CCO/SvO_2_/CEDV; Edwards Lifesciences, Irvine, CA, USA) was performed at the descretion of the treating physician. The site of insertion was confirmed by the transducer waveform, the length of catheter insertion and chest radiography. Systemic arterial pressure was measured invasively using radial or femoral arterial catheterization.

Patients with severe left heart failure due to primary heart disease (left ventricular systolic ejection fraction < 40%, pulmonary artery occlusion pressure > 18 mmHg) were included. The patients were prospectively divided into two groups; group A included patients without severe sepsis or septic shock and group B included patients with additional severe sepsis or septic shock. Severe sepsis and septic shock were defined according to the 1992 American College of Chest Physicians and the Society of Critical Care Medicine consensus conference definitions [12].

All patients received standard treatment for localized infection, severe sepsis and septic or cardiogenic shock, including source control, fluid infusion, catecholamine infusion, replacement and/or support therapy for organ failure, intensive control of blood glucose and corticosteroid substitution therapy, in accordance to current Surviving Sepsis Campaign Guidelines [13]. Mechanically ventilated patients were sedated with midazolam and/or propofol infusion, and no paralytic agents were used.

Fifteen healthy volunteers served as a control group.

### Measurements

#### Skeletal muscle oxygenation

Thenar muscle StO_2 _was measured noninvasively by NIRS (InSpectra™; Hutchinson Technology Inc., Hutchinson, MN, USA). Maximal thenar muscle StO_2 _was determined by moving the probe over the thenar prominence. StO_2 _was continuously monitored and stored in a computer using InSpectra™ software. The average StO_2 _over 15 seconds was used. Measurements were performed immediately after right heart catheterization using pulmonary artery floating catheter insertion (during the first 24 hours after admission). The time between admission and measurement is reported. Measurements in spontaneously breathing patients and healthy volunteers were taken after 15 minutes of bed rest, avoiding any muscular contractions.

#### Severity of disease

Sepsis-related Organ Failure Assessment (SOFA) score was calculated at the time of each measurement to assess the level of organ dysfunction [14]. Dobutamine, norepinephrine requirement represented the dose of drug during the StO_2 _measurement. Also reported is use of intra-aortic balloon pump during ICU stay.

Plasma lactate concentration was measured using enzymatic colorimetric method (Roche Diagnostics GmbH, Mannheim, Germany) at the time of each StO_2 _measurement.

#### Laboratory analysis

Blood was drawn from the pulmonary artery at the time of each StO_2 _measurement in order to determine the SvO_2 _(%). In view of the known problems that may arise during sampling from the pulmonary artery, including the possibility arterial blood may be contaminated with pulmonary capillary blood, all samples from this site were drawn over 30 seconds, using a low-negative pressure technique, and never with the balloon inflated. A standard volume of 1 ml blood was obtained from each side after withdrawal of dead-space blood and flushing fluid. All measurements were made using a cooximeter (RapidLab 1265; Bayer HealthCare AG, Leverkusen, Germany).

### Study of agreement between trends of StO_2 _and SvO_2_

In ten patients from group A and eight patients from group B, StO_2 _and SvO_2 _(Vigilance CEDV; Edwards Lifesciences) were continuously monitored and recorded every 15 minutes for one hour to study agreement between trends in measured variables.

### Data analysis

Data are expressed as mean ± standard deviation. Student's *t*-test, Kolmogorov-Smirnov Z test and χ^2 ^test (Yates correction) were applied to analyze data (SPSS 10.0 for Windows™; SPSS Inc., Cary, NC, USA). One-way analysis of variance with Dunnett T3 test for post-hoc multiple comparisons were used to compare muscle tissue StO_2 _between healthy volunteers and both groups. Spearman correlation test was applied to determine correlation. To compare muscle tissue StO_2 _and SvO_2_, bias, systemic disagreement between measurements (mean difference between two measurements) and precision (the random error in measuring [standard deviation of bias]) were calculated [14]. The 95% limits of agreement were arbitrarily set, in accordance with Bland and Altman [15], as the bias ± 2 standard deviations. *P *< 0.05 (two-tailed) was considered statistically significant.

## Results

Included in the study were 65 patients (36 women and 29 men; mean age 68 ± 14 years) with primary heart disease (ischaemic heart disease in 51 patients, aortic valve stenosis in 12 and dilated cardiomyopathy in two). In 24 patients (group A) severe left heart failure or cardiogenic shock but no additional severe sepsis/septic shock was the reason for ICU admission. In 25 patients severe sepsis and in 16 patients septic shock was diagnosed (group B; *n *= 41). Suspected pneumonia was main source of infection (35 patients [85%]), followed by urinary tract infection (six patients [15%]). In 80% of patients pathogenic bacteria were isolated.

There was no difference in age, sex, aetiology of primary heart disease, echocardiography data, time between admission and measurements, SOFA score, duration of ICU stay and survival between groups (Table 1). Fifteen healthy volunteers (eight women and seven men; age 40 ± 12 years) were included in the control group.

**Table 1:** Description of patients

Parameter	All (*n *= 65)	Group A (*n *= 24)	Group B (*n *= 41)	*P *value
Age (years)	69 ± 15	68 ± 14	70 ± 16	0.2
Female (*n*)	36	12	24	0.9
Ischaemic heart disease (*n*)	51	19	32	0.9
Aoritc stenosis (*n*)	12	4	8	0.9
Dilated cardiomyopathy (*n*)	2	1	1	0.7
LVEF (%)	30 ± 10	28 ± 12	32 ± 8	0.2
LVEDD (cm)	5.8 ± 0.9	5.9 ± 1.0	5.8 ± 0.8	0.3
Severe mitral regurgitation (*n*)	21	8	13	0.9
Time between admission and measurement (hours)	6.4 ± 4.4	6.0 ± 4.8	6.6 ± 4.5	0.6
SOFA score	11.8 ± 2.5	11.6 ± 2.5	11.9 ± 2.7	0.8
ICU stay (days)	8 ± 3	7 ± 4	10 ± 3	0.9
ICU survival (%	47	45	50	0.8

Group A includes patients with severe left heart failure without additional severe sepsis/septic shock, and group B includes patients with severe left heart failure with additional severe sepsis/septic shock. ICU, intensive care unit; LVEDD, left ventricular end-diastolic diameter; LVEF, left ventricular ejection fraction; SOFA, Sequential Organ Failure Assessment.

Patients in group A received higher doses of dobutamine (Table 2). There was no difference in lactate value, haemoglobin level and leucocyte count; however C-reactive protein and procalcitonin values were higher in group B patients (Table 3). Patients in group A had lower cardiac index, DO_2 _and SvO_2_, and higher oxygen extraction ratio compared with patients in group B (Table 4).

**Table 2:** Treatment of patients

Treatment	All (*n *= 65)	Group A (*n *= 24)	Group B (*n *= 41)	*P *value
Norepinephrine (mg/min)	0.048 ± 0.049	0.039 ± 0.042	0.051 ± 0.052	0.39
Dobutamine (mg/min)	0.40 ± 0.31	**0.53 ± 0.33**	**0.33 ± 0.28**	**0.05**
IABP (*n*)	15	**15**	**0**	**0.01**
Mechanical ventilation (*n*)	60	22	38	0.9
FiO_2 _(%)	72 ± 22	**82 ± 19**	**68 ± 23**	**0.04**

Group A includes patients with severe left heart failure without additional severe sepsis/septic shock, and group B includes patients with severe left heart failure with additional severe sepsis/septic shock. FiO_2_, fractional inspired oxygen; IABP, intra-aortic balloon pump.

**Table 3:** Laboratory data

Parameter	All (*n *= 65)	Group A (*n *= 24)	Group B (*n *= 41)	*P *value
Temperature (°C)	37.9 ± 0.9	38.0 ± 0.9	37.9 ± 0.9	0.9
Lactate (mmol/l)	3.5 ± 2.3	4.1 ± 2.5	3.1 ± 2.1	0.1
CRP (mg/l)	110 ± 84	**78 ± 72**	**128 ± 86**	**0.02**
PCT (mg/l)	5.0 ± 6.0	**2.5 ± 2.7**	**6.5 ± 6.8**	**0.02**
Leucocyte (× 10^6^/l)	14.1 ± 7.2	14.5 ± 9.0	13.9 ± 5.2	0.6
Haemoglobin (mg/l)	112 ± 14	109 ± 11	114 ± 15	0.1
Creatinine (μmol/l)	199 ± 165	156 ± 148	227 ± 186	0.1
Arterial blood gas analysis				
pH	7.37 ± 0.03	7.38 ± 0.07	7.36 ± 0.1	0.5
PCO_2 _(kPa)	4.8 ± 1.0	4.7 ± 1.1	4.9 ± 1.0	0.4
PO_2 _(kPa)	16.6 ± 8.0	17.8 ± 10.8	15.9 ± 6.2	0.4
HCO_3 _(mmol/l)	19.8 ± 5.7	**17.1 ± 2.6**	**21.0 ± 6.9**	**0.01**
BE (mEq/l)	-4.8 ± 5.7	**-7.4 ± 3.5**	**-3.6 ± 6.4**	**0.03**
SatHbO_2 _(%)	97 ± 2	97 ± 1	97 ± 3	0.3

Group A includes patients with severe left heart failure without additional severe sepsis/septic shock, and group B includes patients with severe left heart failure with additional severe sepsis/septic shock. BE, base excess; CRP, C-reactive protein; PCO_2_, partial carbon dioxide tension; PCO_2_, partial oxygen tension; PCT, procalcitonin; SatHbO_2_, haemoglobin oxygen saturation.

**Table 4:** Systemic haemodynamics and systemic oxygen transport data

Parameter	All (*n *= 65)	Group A (*n *= 24)	Group B (*n *= 41)	*P *value
Heart rate (beats/min)	111 ± 21	111 ± 24	111 ± 19	0.9
SAP (mmHg)	120 ± 22	122 ± 25	119 ± 22	0.8
DAP (mmHg)	73 ± 21	71 ± 22	74 ± 22	0.7
PAP_s _(mmHg)	56 ± 14	57 ± 13	56 ± 12	0.2
PAP_d _(mmHg)	28 ± 8	**31 ± 8**	**27 ± 8**	**0.01**
CVP (mmHg)	15 ± 4	17 ± 3	14 ± 5	0.051
PAOP (mmHg)	23 ± 6	24 ± 5	22 ± 7	0.9
CI (l/min per m^2^)	2.4 ± 0.7	**2.1 ± 0.6**	**2.6 ± 0.7**	**0.01**
SvO_2 _(%)	63 ± 12	**56 ± 11**	**68 ± 10**	**0.01**
DO_2 _(ml/min per m^2^)	366 ± 134	**301 ± 90**	**404 ± 142**	**0.001**
VO_2 _(ml/min per m^2^)	120 ± 42	125 ± 42	117 ± 41	0.3
O_2_ER (%)	35 ± 12	**43 ± 12**	**31 ± 11**	**0.001**

Group A includes patients with severe left heart failure without additional severe sepsis/septic shock, and group B includes patients with severe left heart failure with additional severe sepsis/septic shock. CI, cardiac index; CVP, central venous pressure; DAP, systemic diastolic artieral pressure; DO_2_, oxygen delivery; O_2_ER, oxygen extraction ratio; PAOP, pulmonary artery occlusion pressure; PAP_d_, pulmonary artery diastolic pressure; PAP_s_, pulmonary artery systolic pressure; SAP, systemic systolic arterial pressure; ScvO_2_, central venous oxygen saturation; SvO_2_, mixed venous oxygen saturation; VO_2_, oxygen consumption.

**Figure 1:**
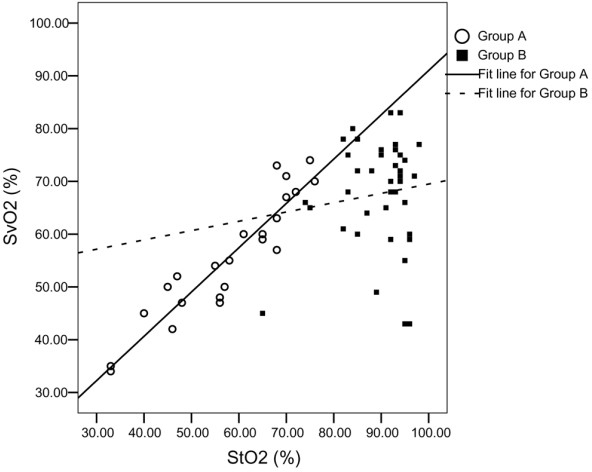
Correlation between skeletal muscle StO_2 _and SvO_2_. Group A includes patients with severe left heart failure without severe sepsis/septic shock, and group B includes patients with primary heart disease and additional severe sepsis/septic shock. A statistically significant correlation was found in group A (*r *= 0.689, *P *= 0.002) but not in group B (*r *= -0.091, *P *= 0.60). StO_2_, tissue oxygenation; SvO_2_, mixed venous oxygen saturation.

**Figure 2:**
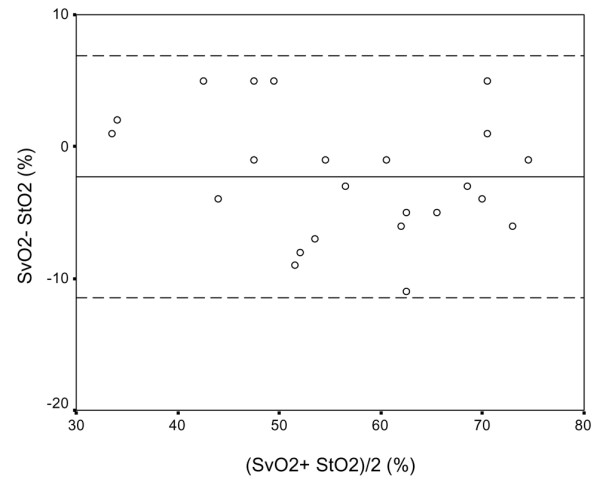
Agreement between SvO_2 _and thenar muscle StO_2 _in the absence of severe sepsis/septic shock. Shown are Bland and Altman plots of agreement between SvO_2 _and thenar muscle StO_2 _in patients with left heart failure without severe sepsis/septic shock (*n *= 24), The unbroken line indicates the mean difference (bias), and broken lines indicate 95% limits of agreement (mean ± standard deviation). StO_2_, tissue oxygenation; SvO_2_, mixed venous oxygen saturation.

In group A StO_2 _was lower than in group B patients and in healthy volunteers (58 ± 13%, 90 ± 7% and 84 ± 4%, respectively; *P *< 0.001). StO_2 _was higher in group B patients than in healthy volunteers (*P *= 0.02). In group A StO_2 _correlated with SvO_2 _(*r *= 0.689, *P *= 0.002), but no correlation was observed between StO_2 _and SvO_2 _in group B (*r *= -0.091, *P *= 0.60; Figure 1). In group A StO_2 _slightly overestimated SvO_2 _(bias -2.3%, precision 4.6%; Figure 2). In group B StO_2 _overestimated SvO_2_, but important disagreement between these variables was observed. In three of our patients with septic shock a skeletal muscle StO_2 _of 75% or lower (lower bound of the 95% confidence interval for mean StO_2 _in control individuals) was detected.

**Figure 3:**
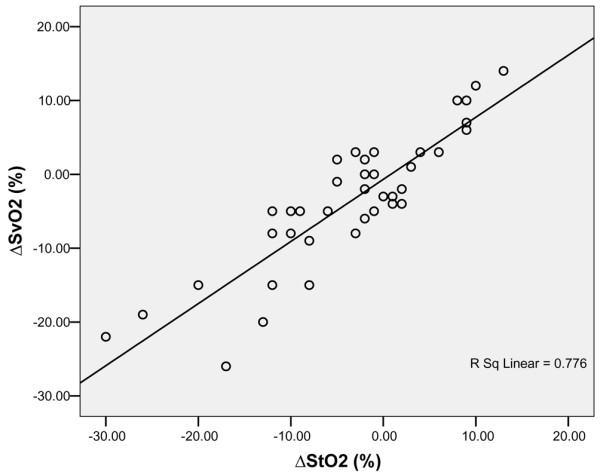
Concordance between changes in SvO_2 _and changes in thenar muscle StO_2 _in the absence of severe sepsis/septic shock. Shown are changes in SvO_2 _and thenar muscle StO_2 _in 10 patients with severe left heart failure without additional severe sepsis/septic shock (group A; *n *= 40, *r *= 0.836, *R*^2 ^= 0.776, *P *< 0.001; equation of the regression line: ΔSvO_2 _[%] = 0.84 × ΔStO_2 _[%] - 0.67). StO_2_, tissue oxygenation; SvO_2_, mixed venous oxygen saturation.

In 10 patients from group A 42 pairs of SvO_2_-StO_2 _changes were recorded. Changes in StO_2 _correlated with changes in SvO_2 _(*r *= 0.836, *R*^2 ^= 0.776, *P *< 0.001); the equation for the regression line was as follows (Figure 3): ΔSvO_2 _(%) = 0.84 × ΔStO_2 _(%) - 0.67. In eight patients from group B 38 pairs of SvO_2_-StO_2 _changes were recorded. In group B changes in StO_2 _did not correlate with changes in SvO_2 _(*r *= 0.296, *R*^2 ^= 0.098, *P *= 0.071).

Plasma lactate concentrations correlated negatively with StO_2 _values in group A (*n *= 24; *r *= -0.522, *P *= 0.009, *R*^2 ^= 0.263; lactate [mmol/l] = -0.104 × StO_2 _[%] + 10.25); there was no correlation between lactate and StO_2 _in group B.

## Discussion

The main result of the study is that skeletal muscle StO_2 _does not estimate SvO_2 _in patients with severe left heart failure and additional severe sepsis or septic shock. However, in patients with severe left heart failure without additional severe sepsis or septic shock, the StO_2 _value could be used as a fast and noninvasive estimate of SvO_2_; also, the trend in StO_2 _may be considered a surrogate for the trend in SvO_2_.

### Skeletal muscle StO_2 _in patients with severe heart failure and additional severe sepsis or septic shock

We previously detected high StO_2 _and slow deceleration in StO_2 _during stagnant ischaemia in septic patients [11]. Our data were in concordance with a previous report from De Blasi and coworkers [16]. Studies in animals and patients with sepsis confirmed the presence of increased tissue oxygen tension [17]. However, tissue oxygen consumption slows down in sepsis, and this correlates with the severity of sepsis [18]. Reduced cellular use/extraction of oxygen may be the problem rather than tissue hypoxia *per se*, because an increase in tissue oxygen tension is normally observed [19]. The high StO_2 _levels seen in our patients with additional severe sepsis or septic shock support this hypothesis. Mitochondrial dysfunction has been implicated by Ince and Sinaasappel [20]. This mitochondrial alteration was also shown to correlate with outcome in sepsis and septic shock [21].

The high StO_2_/low SvO_2_, seen in severe sepsis and septic shock, suggest blood flow redistribution. Thenar muscle StO_2 _probably correlates with central venous oxygen saturation (ScvO_2_), which is measured in a mixture of blood from head and both arms. In healthy resting individuals ScvO_2 _is slightly lower than SvO_2 _[22]. Blood in the inferior vena cava has high oxygen content because the kidneys do not utilize much oxygen but receive a high proportion of cardiac output [23]. As a result, inferior vena caval blood has higher oxygen content than blood from the upper body, and SvO_2 _is greater than ScvO_2_.

This relationship changes in the presence of cardiovascular instability. Scheinman and coworkers [24] performed the earliest comparison of ScvO_2 _and SvO_2 _in both haemodynamically stable and shocked patients. In stable patients ScvO_2 _was similar to SvO_2_. In patients with failing heart ScvO_2 _was slightly higher than SvO_2 _and in shock patients the difference between SvO_2 _and ScvO_2 _was even more pronounced (47.5 ± 15.11% and 58.0 ± 13.05%, respectively; *P *< 0.001). Lee and coworkers [25] described similar findings. Other, more detailed studies in mixed groups of critically ill patients designed to test whether the ScvO_2 _measurements could substitute for SvO_2 _demonstrated problematic large confidence limits [26] and poor correlation between the two values [27].

Most authors attribute this pattern to changes in the distribution of cardiac output that occur in the presence of haemodynamic instability. In shock states, blood flow to the splanchnic and renal circulations falls, whereas flow to the heart and brain is maintained [28]. This results in a fall in the oxygen content of blood in the inferior vena cava. As a consequence, in shock states the normal relationship is reversed and ScvO_2 _is greater than SvO_2 _[23-25]. Consequently, when using ScvO_2 _(or probably StO_2_) as a treatment goal, the relative oxygen consumption of the superior vena cava system may remain stable at a time when oxidative metabolism of vital organs, such as the splanchnic region, may reach a level at which flow-limited oxygen consumption occurs, together with marked decrease in oxygen saturation. In this situation StO_2 _provides a falsely favourable impression of adequate body perfusion, because of the inability to detect organ ischemia in the lower part of the body.

In the present study three patients with septic shock had skeletal muscle StO_2 _of 75% or less (under the lower bound of the 95% confidence interval for the mean StO_2 _in control individuals); they were all in septic shock (lactate value > 2.5 mmol/l) with low cardiac index (< 2.0 l/min per m^2^). These patients were probably in an early under-resuscitated phase of septic shock. Low numbers of septic patients with low StO_2 _values did not allow us to study the agreement between StO_2 _and SvO_2 _in such patients; however, there was a wide range in StO_2 _values with SvO_2 _below 65%. Additional research is necessary to study muscle skeletal StO_2 _in under-resuscitated septic patients.

### Skeletal muscle StO_2 _in patients with severe heart failure without additional severe sepsis or septic shock

Our data are supported by previous work conducted by Boekstegers and coworkers [29], who measured the oxygen partial pressure distribution in biceps muscle. They found low peripheral oxygen availability in cardiogenic shock compared with sepsis. In cardiogenic shock skeletal muscle partial pressure of oxygen correlated with systemic DO_2 _(*r *= 0.59, *P *< 0.001) and systemic vascular resistance (*r *= 0.74, *P *< 0.001). No correlation was found between systemic oxygen transport variables and skeletal muscle partial oxygen pressure in septic patients. These measurements were taken in the most common cardiovascular state in sepsis; this is in contrast to hypodynamic shock, which is only present in the very final stages of sepsis or in patients without adequate volume replacement [30]. In a subsequent study, those authors showed that even in the final state of hypodynamic septic shock, leading to death, the mean muscle partial oxygen pressure did not decrease to under 4.0 kPa before circulatory standstill took place [31].

In a human validation study [32] a significant correlation between NIRS-measured StO_2 _and venous oxygen saturation (*r *= 0.92, *P *< 0.05) was observed; the venous effluent was obtained from a deep forearm vein that drained the exercising muscle. StO_2 _was minimally affected by skin blood flow. Changes in limb perfusion affect StO_2_; skeletal muscle StO_2 _decreases during norepinephrine and increases during nitroprusside infusion.

In shock with preserved or even increased oxygen extraction, such as haemorrhagic shock, StO_2 _(as measured by NIRS in skeletal muscle, stomach and liver) correlated with systemic DO_2 _in a pig model [33]. Changes in skeletal muscle oxygen partial pressure were confirmed during haemorrhagic shock and resuscitation [34]. Continuous monitoring of skeletal muscle StO_2 _is already used in trauma patients, in whom it identifies the severity of shock [35]. Basal skeletal muscle StO_2 _can track systemic DO_2 _during and after resuscitation of trauma patients [36].

StO_2 _overestimated SvO_2 _(bias -2.5%) in the present study. This may be due to the NIRS method, which does not discriminate between compartments. It provides a global assessment of oxygenation in all vascular compartments (arterial, venous and capillary) in sample volume of underlying tissue. This is major limitation of the present study. The noninvasive measurement of only venous oxygen saturation is complicated by the fact that isolation of the contribution of venous compartment to the noninvasive optical signal is not straightforward. New methods like near-infrared spiroximetry, which measures venous oxygen saturation in tissue from the near-infrared spectrum of the amplitude of respiration-induced absorption oscillations, may lead to the design of a noninvasive optical instrument that can provide simultaneous and real-time measurements of local arterial, tissue and venous oxygen saturation [37].

In low flow states, in which controversies regarding monitoring persist [38], it appears logical to make use of both macro- and microcirculatory parameters to guide resuscitation efforts [39]. A large prospective study is currently being performed to evaluate the utility of additional StO_2 _regional monitoring to guide tissue oxygenation, in addition to the early goal-directed therapy proposed by Rivers and coworkers [40].

## Conclusion

In patients with severe left heart failure without additional severe sepsis or septic shock, SvO_2 _provides a noninvasive estimate of and tracks with StO_2_. It should be emphasized that in patients with severe heart failure and additional severe sepsis or septic shock, skeletal muscle StO_2 _provides a falsely favourable impression of body perfusion.

## Key messages

• Skeletal muscle StO_2 _does not estimate SvO_2 _in patients with severe left heart failure and additional severe sepsis or septic shock.

• StO_2 _values could be used to provide rapid, noninvasive estimation of SvO_2_; furthermore, the trend in StO_2 _may be considered a surrogate for the trend in SvO_2_.

## Abbreviations

DO_2 _= oxygen distribution; ICU = intensive care unit; NIRS = near infrared spectroscopy; ScvO_2 _= central venous oxygen saturation; SOFA = Sepsis-related Organ Failure Assessment; StO_2 _= tissue oxygenation; SvO_2 _= mixed venous oxygen saturation.

## Competing interests

The authors declare that they have no competing interest.

## Authors' contributions

MP was responsible for conception and design of the study; for acquisition of data, and its analysis and interpretation; and for drafting the manuscript. HM was responsible for conception and design of the study; for acquisition of data, and its analysis and interpretation; and for drafting the manuscript.
